# Prognostic and Diagnostic Values of Semaphorin 5B and Its Correlation With Tumor-Infiltrating Immune Cells in Kidney Renal Clear-Cell Carcinoma

**DOI:** 10.3389/fgene.2022.835355

**Published:** 2022-04-11

**Authors:** Junping Ding, Shubin Zhao, Xianhua Chen, Changjun Luo, Jinjian Peng, Jiantan Zhu, Yongqi Shen, Zhou Luo, Jianlin Chen

**Affiliations:** ^1^ Departments of Urology of Affiliated Liutie Central Hospital of Guangxi Medical University, Liuzhou, China; ^2^ Departments of Clinical Laboratory, Key Laboratory of Medical Molecular Diagnostics of Liuzhou, Key Laboratory for Nucleic Acid Molecular Diagnosis and Application of Guangxi Health & Wellness Commission, Affiliated Liutie Central Hospital of Guangxi Medical University, Liuzhou, China; ^3^ Departments of Cardiology of Affiliated Liutie Central Hospital of Guangxi Medical University, Liuzhou, China; ^4^ Departments of Oncology of Affiliated Liutie Central Hospital of Guangxi Medical University, Liuzhou, China; ^5^ Departments of Infectious Diseases of Affiliated Liutie Central Hospital of Guangxi Medical University, Liuzhou, China

**Keywords:** sema5b, KIRC, diagnostic, biomarker, prognosis

## Abstract

**Background:** Semaphorin 5B (SEMA5B) has been described to be involved in the development and progression of cancer. However, the potential diagnostic and prognosis roles and its correlation with tumor-infiltrating immune cells in KIRC have not been clearly reported yet.

**Methods:** The mRNA level of SEMA5B was analyzed via the TCGA and GTEx database as well as the CCLE dataset and verified by GSE53757 and GSE40435 datasets. Meanwhile, the protein level of SEMA5B was analyzed by CPTAC and validated by HPA. The diagnostic value of SEMA5B was analyzed according to the TCGA database and validated by GSE53757, GSE46699, and GSE11024 + GSE46699 datasets. Then, the survival analysis was conducted using GEPIA2. R software (v3.6.3) was applied to investigate the relevance between SEMA5B and immune checkpoints and m6A RNA methylation regulator expression. The correlation between SEMA5B and MMRs and DNMT expression and tumor-infiltrating immune cells was explored via TIMER2. Co-expressed genes of SEMA5B were assessed by cBioPortal, and enrichment analysis was conducted by Metascape. The methylation analysis was conducted with MEXPRESS and MethSurv online tools. Gene set enrichment analysis (GSEA) was applied to annotate the biological function of SEMA5B.

**Results:** SEMA5B was significantly upregulated at both the mRNA and protein levels in KIRC. Further analysis demonstrated that the mRNA expression of SEMA5B was significantly correlated with gender, age, T stage, pathologic stage, and histologic grade. High levels of SEMA5B were found to be a favorable prognostic factor and novel diagnostic biomarker for KIRC. SEMA5B expression was shown to be significantly associated with the abundance of immune cells in KIRC. Also, SEMA5B expression was significantly correlated with the abundance of MMR genes, DNMTs, and m6A regulators in KIRC. Enrichment analysis indicated that the co-expressed genes may involve in crosslinking in the extracellular matrix (ECM). GSEA disclosed that SYSTEMIC_LUPUS_ERYTHEMATOSUS and NABA_ECM_REGULATORS were prominently enriched in the SEMA5B low-expression phenotype. Finally, the methylation analysis demonstrated a correlation between hypermethylation of the SEMA5B gene and a poor prognosis in KIRC.

**Conclusion:** Increased SEMA5B expression correlated with immune cell infiltration, which can be served as a favorable prognostic factor and a novel diagnostic biomarker for KIRC.

## Introduction

Renal cell carcinoma (RCC) is the third most common malignancy in the urological system (Siegel et al., 2018) and ranks in the top 10 most common malignancies worldwide, accounting for nearly 3% of all malignancies (Sigel et al., 2002). Kidney renal clear cell carcinoma (KIRC) is the main histology type of RCC, accounting for 80% of tumors (Shuch et al., 2015). In the past few decades, although significant progress has been made in the treatment of KIRC, the 5-year overall survival rate (OS) for patients with advanced stages is still less than 10% (Hsieh et al., 2017). Currently, surgery is the mainstay of treatment for KIRC, but is associated with a high rate of recurrence and distant metastasis (Chin et al., 2006). Chemotherapy and radiotherapy were the main adjuvant treatment after operation in the past decade; both of them have been proven to exhibit poor prognosis (Ravaud et al., 2016; Gao et al., 2020). In recent years, immunotherapy has shown good clinical effects in the treatment of renal cancer and has become a research hotspot in this field. However, immunotherapy has a low rate of reaction owing to a lack of sensitive and specific biomarkers (Atkins, 2009). Therefore, it is necessary to find more effective and safer therapeutic targets to improve the survival outcome of ccRCC patients.

In 1993, Kolodkin originally identified and characterized that semaphorins are the family of phylogenetically conserved molecules which play some roles in providing attractive and repulsive axon guidance cues during axon growth (Schwab, 1996). A previous study found that the Semaphorin 4D can affect tumor progression by various mechanisms, including modulation of tumor angiogenesis (Basile et al., 2007). Semaphorin 5B (SEMA5B) is a member of semaphorins. The knowledge about SEMA5B is mostly acquired from the studies of mouse retinas and chicken spinal cords, where SEMA5B uses plexina1 and plexina3 signals as rejection cues (Matsuoka et al., 2011; Liu et al., 2014). In recent years, increasing evidence has indicated the pivotal role of SEMA5B involved in the pathogenesis or progression of tumors. SEMA5B has been identified as a candidate gene for changes associated with asbestos exposure in pulmonary disease studies (Mäki-Nevala et al., 2016). WU et al. also found that SEMA5B can be considered as a candidate gene for a susceptibility locus in esophageal cancer (Wu et al., 2014). In addition, SEMA5B acts as a potential prognostic marker in gastric carcinoma (Cao et al., 2021). However, it was not until 2009 that researchers first demonstrated the expression of SEMA5B in human kidney (Cuellar, 2009). A few recent studies disclosed that downregulation SEMA5B expression attenuated the RCC cell viability (Hirota et al., 2006), while increased SEMA5B expression promoted proliferation in HK2 cells (Kundu et al., 2020). Moreover, RT-qPCR confirmed that SEMA5B expression was significantly elevated in ccRCC, and SEMA5B was identified as a target gene of HIF that promoted tumor growth *in vivo* (Kundu et al., 2020). However, to our best knowledge, the potential diagnostic and prognosis roles as well as the underlying mechanism of SEMA5B in KIRC have not been clearly described yet.

In the current study, we carried out a bioinformatics analysis and primarily aimed to comprehensively explore the expression level, diagnostic values, prognostic values, and gene methylation characteristics of SEMA5B in KIRC. Next, the correlation between SEMA5B expression and the immune environment was also explored. Finally, we identified and analyzed the co-expressed genes of SEMA5B in KIRC. This study provides a comprehensive insight into the underlying significance of SEMA5B and would lead to a better understanding of the possible role of SEMA5B in tumor immunology and its clinical value in KIRC.

## Materials and Methods

### Expression Analyses of *SEMA5B*

Based on the TCGA database, we employed GEPIA2 (http://gepia2.cancer-pku.cn/#analysis) to assess the gene expression profiles of SEMA5B among 33 cancer types. Therefore, we investigated the expression level of SEMA5B gene between KIRC and normal tissues in TCGA datasets alone or the combination of TCGA with GTEx databases (https://www.gtexportal.org/home/-index.html). Furthermore, RNA-sequencing and clinical follow-up (overall survival [OS] and disease-free survival [DFS]) data of 539 ccRCC and 72 adjacent normal tissues were downloaded from TCGA, and the expression level of SEMA5B in 72 paired samples was determined. The log2 [TPM (Transcripts per million) +1] or log2 [FPKM (Fragments Per Kilo base per Million) +1] transformed expression data was applied for data analysis. We also explored the expression level of the protein of SEMA5B between primary tumor and normal tissues via the CPTAC (Clinical proteomic tumor analysis consortium) dataset. The immunohistochemical data of SEMA5B protein expression in KIRC and normal tissue was obtained from the Human Protein Atlas (HPA) (https://www.proteinatlas.org/). The microarray data of GSE53757 and GSE40435 were obtained from the Gene Expression Omnibus (GEO) database (https:// www.ncbi.nlm.nih.gov/geo/) and used as validation KIRC datasets, and gene expression profiles were identified using the edgeR package (Smyth, 2010) in R (v3.6.3). The cell line expression matrix of 32 tumors was obtained from the CCLE dataset (https://portals.broadinstitute.org/ccle/about). Gene expression profiles were constructed by the R v4.0.3 software package ggplot2 (v3.3.3) (Ghandi et al., 2019).

### Diagnostic, Survival, and Prognosis Analysis

We used the “Survival Analysis” module of GEPIA2 to plot the OS (overall survival) and DFS (disease-free survival) survival curves in KIRC with the “Median” as the expression thresholds for splitting the high-expression and low-expression cohorts. The correlation between SEMA5B expression and OS, RFS (recurrence-free survival) in KIRC were also analyzed by Kaplan–Meier plotter (http://kmplot. com/analysis/) (Lánczky et al., 2016). The hazard ratio (HR) with 95% confidence intervals and log-rank *p*-value were also computed. The diagnostic values of SEMA5B was calculated by the pROC package (Robin et al., 2011) of R version 3.6.3, and the ROC curves were visualized by the ggplot2 package (Wickham, 2016). The prognosis values of SEMA5B and clinical features were calculated by univariate and multivariate Cox regression analysis. The R package “randomForest” was used for random forest regression.

### Association Between SEMA5B Expression and Clinical Features

After 539 KIRC cases with complete clinical data obtained, the correlation between clinical features and SEMA5B expression were analyzed on R. Wilcoxon rank sum or Kruskal–Wallis rank sum tests were used for significance tests.

### Analyses of Genes Co-expressed With SEMA5B in KIRC

First, we identified the 300 co-expressed genes of SEMA5B by “Co-expression” module of cBioPortal, selected the top six co-expressed genes of SEMA5B according to *p* values, and visualized the correlation analysis in the cBioPortal. Then, we conducted the survival analysis of top six co-expressed genes via the GEPIA2. In addition, we performed the enrichment analysis (Gene Ontology (GO) and the Kyoto Encyclopedia of Genes and Genomes (KEGG) pathway) of co-expressed genes of SEMA5B using the Metascape (Zhou et al., 2019).

### Association of SEMA5B and Immune Cell Infiltration in KIRC

TIMER2 (https://cistrome.shiny apps. io/timer/) database was used to systematically explore the correlations between SEMA5B expression and the tumor-infiltrating immune cells (TIICs).

### Correlation Analysis Between SEMA5B and MMR Genes and DNA Methyltransferase Analysis

TIMER database was used to assess the correlations between SEMA5B expression and DNA mismatch repair (MMR) genes (MLH1, MSH2, MSH6, PMS2, and EPCAM), DNA methyltransferase genes (DNMT1, DNMT2, DNMT3A, and DNMT3B). GEPIA was used to verify the gene correlation analysis in the TIMER database.

### Correlation Analysis Between SEMA5B and Immune Checkpoint Gene Analysis

The expression levels of 8 immune checkpoint-related genes (SIGLEC15, TIGIT, CD274, HAVCR2, PDCD1, CTLA4, LAG3, and PDCD1LG2) were detected, and the R 3.6.3 programming language was used to perform the statistical analysis.

### DNA Methylation Analysis

The MEXPRESS tool was used to analyze the SEMA5B methylation corresponding to TCGA–KIRC cohorts. Also, the Pearson correlation analyses between DNA methylation and SEMA5B expression were performed. The MethSurv tool was used to visualize SEMA5B methylation and the Kaplan–Meier based correlation between SEMA5B hyper/hypomethylation and OS.

### Correlations Between SEMA5B Expression and m6A RNA Methylation Regulators

Differentially expressed m6A RNA methylation regulators (KIRCs vs normal tissues, high- and low-SEMA5B KIRCs) were analyzed by the Mann–Whitney *U* test method in R (version R 3.6.3). ns, *p* ≥ 0.05; *, *p* < 0.05; **, *p* < 0.01; ***, *p* < 0.001.

### Gene Set Enrichment Analysis (GSEA)

GSEA (Subramanian et al., 2005) analysis was used to identify functional and biological pathways between low and high expression of SEMA5B from TCGA gene expression data. The expression level of SEMA5B was served as a phenotype label. Normalized enrichment scores (NES) were acquired by analyzing genes with permutations 1,000 times. The statistical significance of pathways is dependent on normal *p* < 0.05 and false discovery rate (FDR) q<0.05.

## Results

### The Expression Profiles of SEMA5B Were Upregulated in KIRC

**FIGURE 1:**
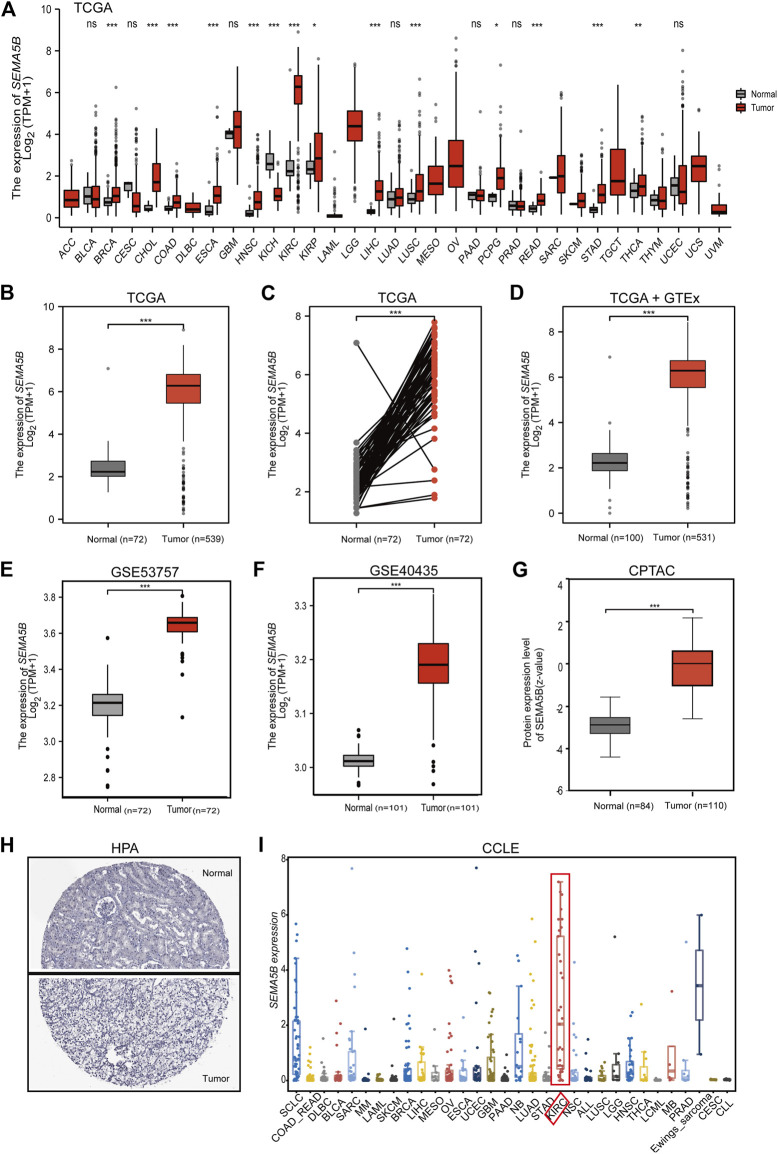
The mRNA and protein level of SEMA5B in the KIRC tissue and KIRC cell lines. **(A)** The comparison of SEMA5B expression between tumor and noncancerous adjacent tissues in different types of cancers based on the TCGA database. **(B)** The expression of SEMA5B in normal and tumor tissues based on the TCGA. **(C)** The expression of SEMA5B in paired tissues based on the TCGA. **(D)** The expression of SEMA5B in normal and tumor tissues based on matching TCGA normal and GTEx data. The expression of SEMA5B in KIRC based on GSE53757 **(E)** and GSE40435 **(F)**. **(G)** Protein expression levels of SEMA5B in normal and KIRC tumor tissues based on the CPTAC dataset. **(H)** Representative immunohistochemistry images of SEMA5B in KIRC and normal tissues derived from the HPA database. **(I)** The mRNA expression levels of SEMA5B in kidney cancer cell lines using the CCLE. ns, *p* ≥ 0.05; *p < 0.05; **p < 0.01; ***p < 0.001.

Based on the TCGA database, we explored the expression profiles of SEMA5B in pan-cancers by GEPIA2. As shown in Figure 1A, the mRNA expression of SEMA5B was overexpressed in tumors than normal tissues in different types of cancer. Especially, SEMA5B was found to be highly expressed in KIRC than normal tissues (Figure 1B, *p* < 0.001) and paired adjacent normal tissues (Figure 1C, *p* < 0.001). Including the normal tissue of the GTEx dataset as controls for further analysis, SEMA5B was still highly expressed in KIRC than normal tissues (Figure 1D, *p* < 0.001). Furthermore, the online Gene Expression Omnibus (GEO) database showed consistent results based on the data from the GSE53757 and GSE40435 datasets (Figure 1 E, F, all *p* < 0.001). Then, we analyzed the protein expression of SEMA5B from the CPTAC databases. More importantly, the protein expression of SEMA5B was also elevated in tumor samples when compared to adjacent non-tumor samples (Figure 1G, *p* < 0.001). Moreover, the IHC staining results from the HPA database further confirmed the significantly higher protein levels of SEMA5B in tumor tissues (Figure 1H). We also performed the expression levels of SEMA5B in RCC cell lines using the Cancer Cell Line Encyclopedia (CCLE) of the Broad Institute. Our results showed that SEMA5B was significantly upregulated in RCC cell lines than other cancer types (Figure 1I). Collectively, all these data strongly confirmed the high-expression status of SEMA5B in KIRC.

### The Expression Level of SEMA5B Was Associated With the Clinicopathological Characteristics of KIRC Patients

**FIGURE 2:**
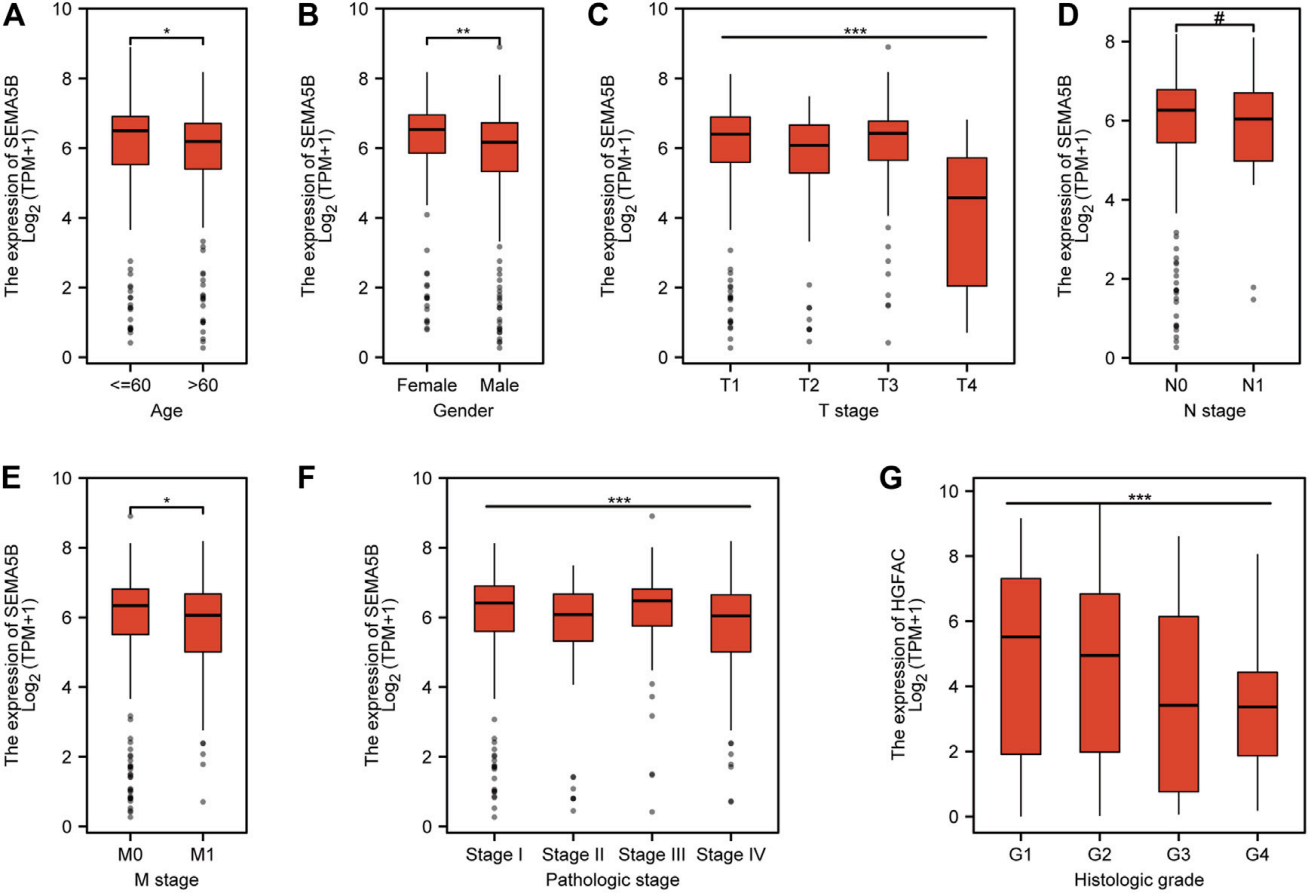
Associations of SEMA5B expression with clinical parameters. **(A)** Age (*p* < 0.05); **(B)** Gender (*p* < 0.01); **(C)** T stage (*p* < 0.001); **(D)** N stage (*p* < 0.05); **(E)** M stage (*p* < 0.05); **(F)** Pathologic stage (*p* < 0.001); and **(G)** histologic grade (*p* < 0.001).

Since the function of SEMA5B in KIRC is still unclear, a way to further analyze the relationships between SEMA5B expression and some clinical parameters in KIRC patients is necessary. We separated the patients into high- and low-SEMA5B mRNA-expressing groups based on the median SEMA5B mRNA expression. Remarkably, SEMA5B expression was associated with gender (*p* = 0.003), age (*p* = 0.018), T stage (*p* = 0.019), pathologic stage (*p* = 0.036), and histologic grade (*p* = 0.006) (Table 1). However, there was no significant difference in the distribution of clinical N stages (*p* = 0.488) and M stage (*p* = 0.188). We conducted additional analyses to further examine the association of the expression of SEMA5B with clinicopathological parameters in KIRC from TCGA samples. As shown in Figure 2, high expression of SEMA5B was significantly correlated with age (*p* < 0.05), gender (*p* < 0.01), T stage (*p* < 0.001), M stage (*p* < 0.05), pathologic stage (*p* < 0.001), and histologic grade (*p* < 0.001). However, high expression of SEMA5B was not significantly correlated with N stage (*p* > 0.05).

**TABLE 1:** Relationship between the clinical features and SEMA5B expression in patients with KIRC.

Characteristic	Low expression of SEMA5B,n (%)	High expression of SEMA5B,*n* (%)	χ^2^	P
N	269	270		
Gender			8.75	**0.003**
Female	76 (14.1%)	110 (20.4%)		
Male	193 (35.8%)	160 (29.7%)		
Age			5.61	**0.018**
<=60	120 (22.3%)	149 (27.6%)		
>60	149 (27.6%)	121 (22.4%)		
T stage			9.9	**0.019**
T1	132 (24.5%)	146 (27.1%)		
T2	44 (8.2%)	27 (5%)		
T3	84 (15.6%)	95 (17.6%)		
T4	9 (1.7%)	2 (0.4%)		
N stage			0.48	0.488
N0	121 (47.1%)	120 (46.7%)		
N1	10 (3.9%)	6 (2.3%)		
M stage			1.73	0.188
M0	209 (41.3%)	219 (43.3%)		
M1	45 (8.9%)	33 (6.5%)		
Pathologic stage			8.54	**0.036**
Stage I	127 (23.7%)	145 (27.1%)		
Stage II	36 (6.7%)	23 (4.3%)		
Stage III	55 (10.3%)	68 (12.7%)		
Stage IV	49 (9.1%)	33 (6.2%)		
Histologic grade			12.5	**0.006**
G1	2 (0.4%)	12 (2.3%)		
G2	112 (21.1%)	123 (23.2%)		
G3	101 (19%)	106 (20%)		
G4	47 (8.9%)	28 (5.3%)		

Note: Data in bold indicates *p* < 0.05.

### Predictive Value of SEMA5B for KIRC Diagnosis and Prognosis

**FIGURE 3:**
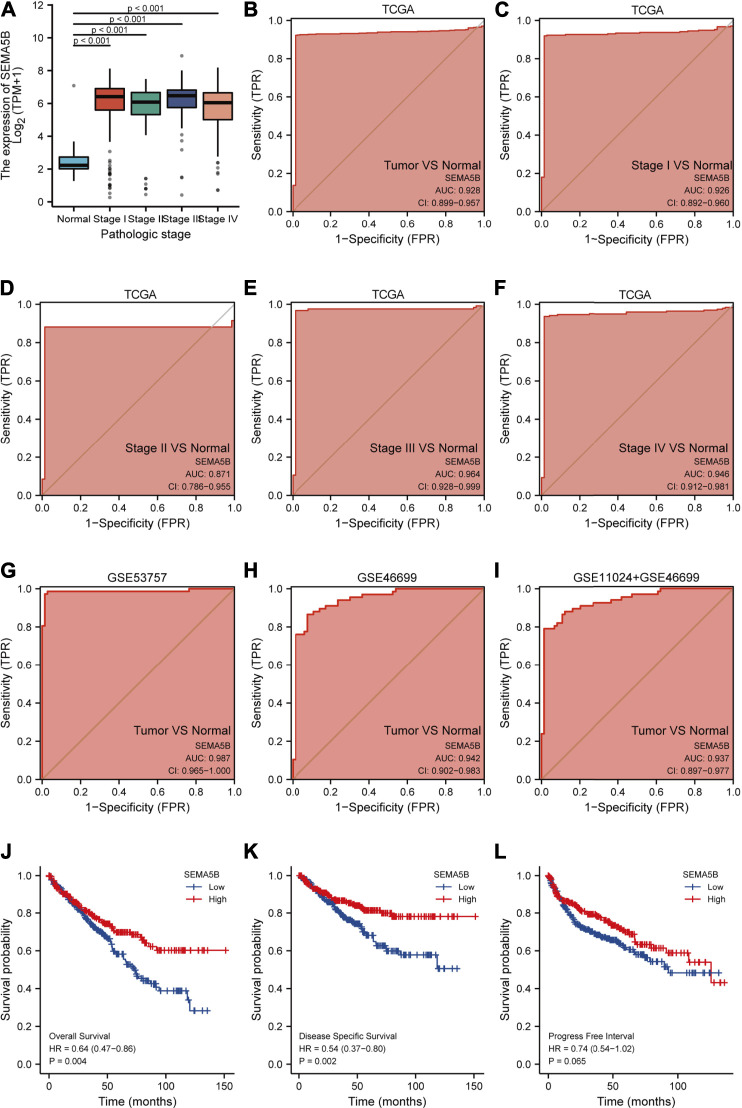
Predictive value of SEMA5B expression for diagnosis and clinical outcomes in KIRC. **(A)** SEMA5B expression is higher in each pathologic stages of KIRC than normal tissue based on the TGCA dataset (all *p* < 0.001); ROC curves of SEMA5B for KIRC cases with overall tumors **(B)**, stage I **(C)**, stage II **(D)**, stage III **(E)**, and stage IV in TCGA datasets; ROC curve in validation set of GSE 53757 **(G)**, GSE46699 **(H)**, and GSE11024 + GSE46699 **(I)** for discriminating overall tumors from normal. Shown are the Kaplan–Meier analyses comparing overall survival **(J)**, disease-specific survival **(K)**, and progression-free interval **(L)** between high- and low-SEMA5B expression groups.

ROC curve is routinely used to evaluate the diagnostic value of the biomarkers in clinics. We then demonstrated the potential for using SEMA5B as a diagnostic biomarker for KIRC; ROC curves and the area under the curve (AUC) were calculated. As shown in Figure 3B, the ROC curve for SEMA5B exhibited an area under the curve (AUC) of 0.928 for distinguishing clear cell RCC. As the high expression of SEMA5B had a trend to be associated with pathologic stage in KIRC patients (Figure 3A), we hypothesized that it could be a better early diagnostic parameter for KIRC. As the AUCs were 0.926,0.871,0.964, and 0.946, SEMA5B showed significantly high sensitivity and specificity in discriminating power of disease stage for the early-stage and late-stage KIRC samples (Figures 3C–F). Thus, the diagnostic capability of SEMA5B was further verified in GSE53757, GSE46699, and GSE11024 + GSE46699 datasets. The AUC values for SEMA5B in discriminating KIRC patients from healthy controls with a AUC of 0.987(95% CI 0.965–1.000), 0.942(95% CI 0.902–0.983), and 0.937 (95% CI 0.897–0.977), respectively. Overall, these results illustrated the potential value of SEMA5B gene as a powerful biomarker for KIRC diagnosis.

Next, we used the K-M analyses of SEMA5B to test its ability to predict clinical outcomes. As can be seen from Figure Figure3J-3K, SEMA5B-high group demonstrated significantly better overall survival and disease-specific survival when compared with low group in KIRC (all *p* < 0.05). However, there were no statistical differences in progression-free interval between the high- and low-SEMA5B groups (Figure3L, *p* > 0.05).

Moreover, we performed a Cox regression univariable and multivariable analysis of characteristics potentially associated with clinical outcomes to further evaluate the predictive value of SEMA5B on overall survival (OS), disease-specific survival (DSS), and progression-free interval (PFI). As shown in Table 2, in the univariate Cox regression analysis, pathologic stage, T stage, N stage, M stage, histologic grade, and hemoglobin were the prognostic factors for OS, DSS, and PFI. Low SEMA5B expression was a risk factor for overall survival (HR: 1.611, *p* < 0.01) and disease-specific survival (HR: 1.896, *p* < 0.01), although it did not provide any significant predictive ability for progression-free interval. Notably, the result of multivariate Cox regression revealed that M stage was an independent risk factor for OS (HR: 2.767, *p* < 0.001), DSS (HR: 3.840, *p* < 0.001) and PFI (HR: 6.751, *p* < 0.001).

**TABLE 2:** Cox regression analysis for clinical outcomes in KIRC patients.

Characteristics	HR for OS (95% CI)		HR for DSS (95% CI)		HR for PFI (95% CI)	
	Univariate	Multivariate	Univariate	Multivariate	Univariate	Multivariate
Gender (male vs. female)	0.930 (0.682–1.268)		1.220 (0.807–1.845)		1.515* (1.067–2.151)	1.462 (0.917–2.331)
Age (≤60 vs. >60years)	0.567*** (0.417–0.770)	0.718 (0.458–1.128)	0.749 (0.513–1.094)		0.784 (0.574–1.071)	
Pathologic stage (stage III–IV vs. stage I–II)	3.946*** (2.872–5.423)	1.030 (0.365–2.909)	9.835 *** (5.925–16.325)	2.146 (0.657–7.004)>	6.817 *** (4.770–9.744)	2.273 (0.848–6.093)
T stage (T3–T4 vs. T1–T2)	3.228 *** (2.382–4.374)	1.882 (0.754–4.698)	5.542 *** (3.652–8.411)	1.798 (0.709–4.557)	4.522 *** (3.271–6.253)	1.683 (0.738–3.840)
N stage (N1 vs. N0)	3.453*** (1.832–6.508)	1.017 (0.439–2.356)	3.852*** (1.825–8.132)	0.998 (0.422–2.361)	3.682 *** (1.891–7.167)	0.755 (0.330–1.723)
M stage (M1 vs. M0)	4.389*** (3.212–5.999)	2.767 *** (1.551–4.936)	9.108 *** (6.209–13.361)	3.840 *** (2.034–7.249)	8.968 *** (6.464–12.442)	6.751 *** (3.600–12.659)
Histologic grade (G3–G4 vs. G1–G2)	2.702*** (1.918–3.807)	1.413 (0.831–2.402)	4.793 *** (2.889–7.952)	1.475 (0.715–3.043)	3.646 *** (2.503–5.310)	1.262 (0.719–2.214)
Hemoglobin (normal vs. abnormal)	0.417 *** (0.292–0.595)	0.582 * (0.355–0.955)	0.399*** (0.254–0.627)	0.718 (0.399–1.291)	0.572 *** (0.403–0.811)	0.955 (0.582–1.567)
SEMA5B (low vs. high)	1.611** (1.187–2.186)	1.313 (0.829–2.079)	1.896 ** (1.277–2.816)	1.600 (0.912–2.810)	1.344 (0.982–1.840)	1.341 (0.829–2.171)

Note: Data in bold indicates *p* < 0.05. HR, hazard ratio; OS, overall survival; DSS, disease-specific survival; PFI, progression-free interval; CI, confidence interval. *p < 0.05; **p < 0.01; ***p < 0.001.

### Analyses of Genes Co-Expressed With SEMA5B in KIRC

**FIGURE 4:**
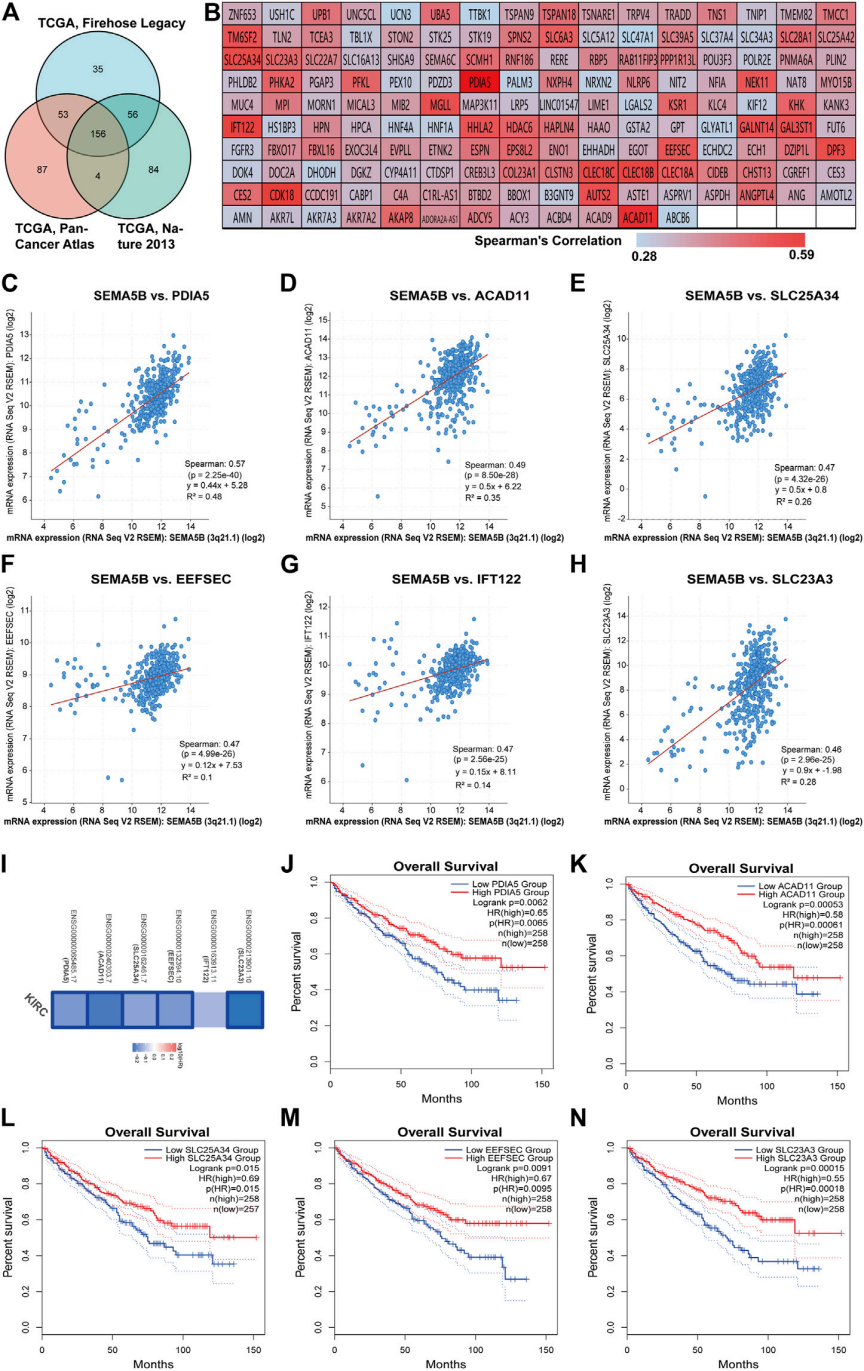
Analyses of genes co-expressed with SEMA5B. **(A)** The Venn diagram of the 156 co-expressed genes in three TCGA studies on KIRC. **(B)** The heat map of the correlation between SEMA5B and its 156 co-expressed genes according to the values of Spearman’s rank correlation coefficient. **(C–H)** Scatterplot showing the correlation between SEMA5B and its top six co-expressed genes. **(I)** Survival map of the six co-expressed genes based on the TCGA–KIRC project data was generated with the GEPIA2 online tool. Blue boxes indicate statistically significant genes. **(J–N)** The K-M (Kaplan–Meier) plots of overall survival (OS) shows the difference between the low- and high-expression of **(J)** PDIA5, **(K)** ACAD11, **(L)** SLC25A34, **(M)** EEFSEC, and **(N)** SLC23A3 in KIRC.

Genes with similar expression profiles (co-expressed genes) are often functionally related, and consequently, co-expression analysis is a robust method for gene function prediction. We applied the c-BioPortal web server to identify the top 300 co-expressed genes with SEMA5B in three different studies from TCGA (TCGA, Firehose Legacy; TCGA, Nature 2013; TCGA, PanCancer Atlas). As shown in Figure 4A, 156 SEMA5B positively co-expressed genes were obtained, which were duplicate genes in three TCGA studies. The values of Spearman’s correlation were shown in Figure 4B. The top six co-expressed genes of SEMA5B arranged by adjusted *p* values were identified. The correlation analysis revealed that SEMA5B was highly positively correlated with protein disulfide-isomerase A5 (PDIA5), acyl-CoA dehydrogenase family member 11 (ACAD11), solute carrier family 25 member 34 (SLC25A34), selenocysteine-specific elongation factor (EEFSEC), intraflagellar transport protein 122 homolog (IFT122), and solute carrier family 23 member 3 (SLC23A3) (Figures 4C–H). The survival map indicated that all six genes were prognostic factors for favorable survival of KIRC, among which five genes showed statistically significant relationships with survival (Figure 4I). Overall survival analysis further confirmed that high expression levels of PDIA5, ACAD11, SLC25A34, EEFSEC, and SLC23A3 were associated with favorable prognosis of KIRC (Figure 4J-N).

**FIGURE 5:**
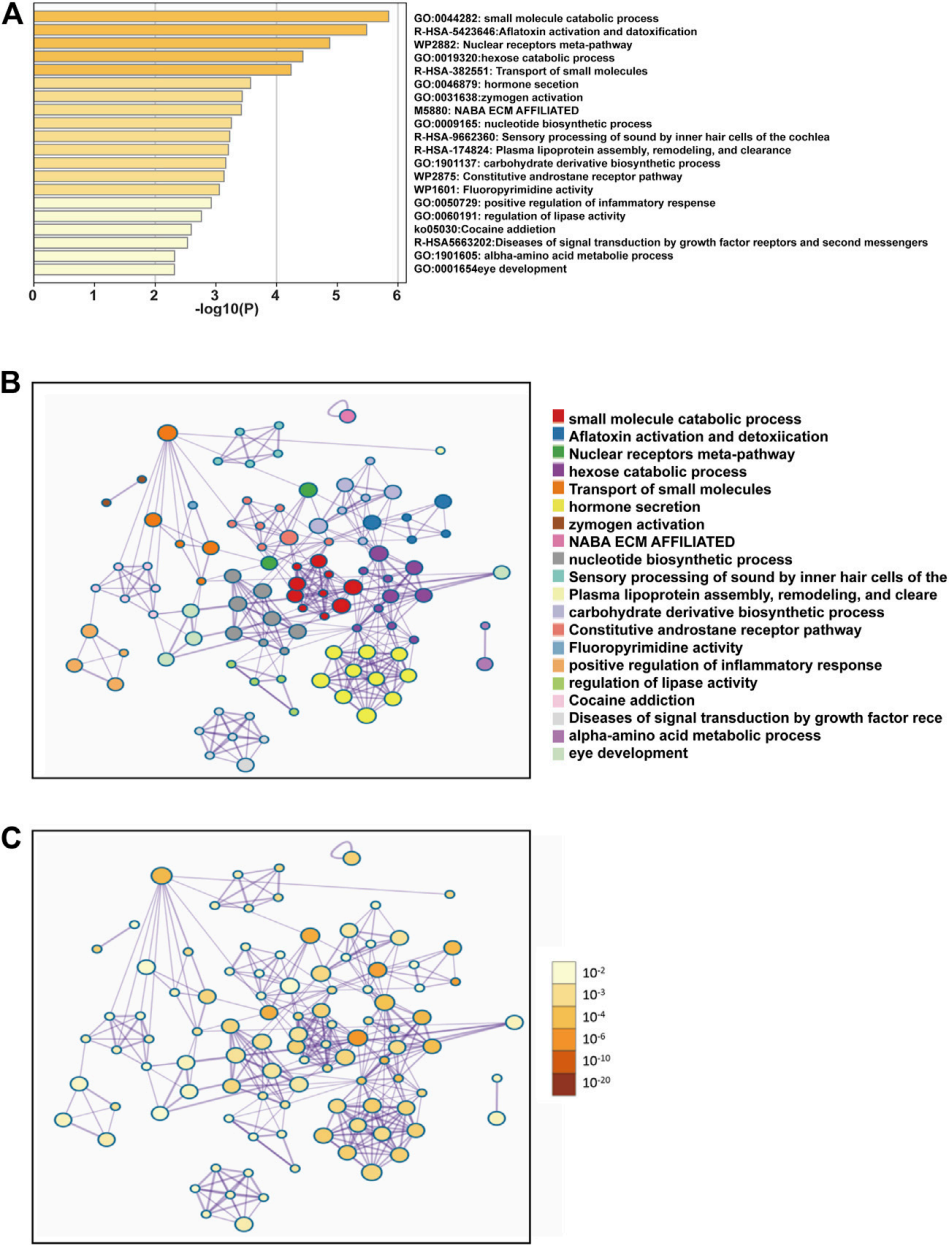
Functional enrichment analysis of the co-expressed genes of SEMA5B. **(A)** Bar graph of enriched terms across co-expressed genes constructed by Metascape, colored by *p*-values. **(B)** Interactive network of the top 20 enriched terms colored by cluster ID. **(C)** Interactive network of the top 20 enriched terms colored by *p*-values.

To further explore the enrichment function of co-expressed genes of SEMA5B, 156 SEMA5B positively co-expressed genes were selected to conduct the Gene Ontology (GO) and Kyoto Encyclopedia of Genes and Genomes (KEGG) pathway analyses through the Metascape. As can be seen from Figure 5A and Table 3, co-expressed genes of SEMA5B were closely enriched in small molecule catabolic process, transport of small molecules, positive regulation of inflammatory response, and diseases of signal transduction by growth factor receptors and second messengers. KEGG pathway analysis identified enrichment for several KIRC-related pathways, including nuclear receptors meta-pathway, NABA ECM AFFILIATED, and the constitutive and rostane receptor pathway. The networks of enrichment terms of SEMA5B colored by cluster ID and *p* value were displayed in Figures 5B,C. Together, these data strongly suggest that SEMA5B may play a crucial role in KIRC through crosslinking in the extracellular matrix (ECM).

**TABLE 3:** Top 20 clusters with their representative enriched terms (one per cluster).

GO	Category	Description	Count	%	Log10(P)	Log10(q)
GO:0044282	GO biological processes	Small-molecule catabolic process	12	7.69	−5.85	−1.48
R-HSA-5423646	Reactome gene sets	Aflatoxin activation and detoxification	4	2.56	−5.48	−1.47
WP2882	WikiPathways	Nuclear receptor meta-pathway	10	6.41	−4.87	−1.11
GO:0019320	GO biological processes	Hexose catabolic process	4	2.56	−4.43	−0.85
R-HSA-382551	Reactome gene sets	Transport of small molecules	14	8.97	−4.24	−0.78
GO:0046879	GO biological processes	Hormone secretion	8	5.13	−3.57	−0.38
GO:0031638	GO biological processes	Zymogen activation	4	2.56	−3.43	−0.38
M5880	Canonical pathways	NABA ECM AFFILIATED	6	3.85	−3.42	−0.38
GO:0009165	GO biological processes	Nucleotide biosynthetic process	7	4.49	−3.25	−0.35
R-HSA-9662360	Reactome gene sets	Sensory processing of sound by inner hair cells of the cochlea	4	2.56	−3.23	−0.35
R-HSA-174824	Reactome gene sets	Plasma lipoprotein assembly, remodeling, and clearance	4	2.56	−3.20	−0.35
GO:1901137	GO biological processes	Carbohydrate derivative biosynthetic process	11	7.05	−3.16	−0.34
WP2875	WikiPathways	Constitutive androstane receptor pathway	3	1.92	−3.13	−0.34
WP1601	WikiPathways	Fluor pyrimidine activity	3	1.92	−3.06	-0.34
GO:0050729	GO biological processes	Positive regulation of inflammatory response	5	3.21	-2.92	−0.25
GO:0060191	GO biological processes	Regulation of lipase activity	4	2.56	−2.76	−0.20
ko05030	KEGG pathway	Cocaine addiction	3	1.92	−2.59	−0.08
R-HSA-5663202	Reactome gene sets	Diseases of signal transduction by growth factor receptors and second messengers	8	5.13	−2.53	−0.05
GO:1901605	GO biological processes	Alpha-amino acid metabolic process	5	3.21	−2.32	0.00
GO:0001654	GO biological processes	Eye development	7	4.49	−2.32	0.00

“Count” is the number of genes in the provided lists with membership in the given ontology term. “%" is the percentage of all of the provided genes that are found in the given ontology term (only input genes with at least one ontology term annotation are included in the calculation). “Log10(P)" is the *p* value in log base 10. “Log10(q)" is the multi-test adjusted *p* value in log base 10.

### Correlation Analysis of SEMA5B Expression and Immune Infiltration of Immune Cells in KIRC

**FIGURE 6:**
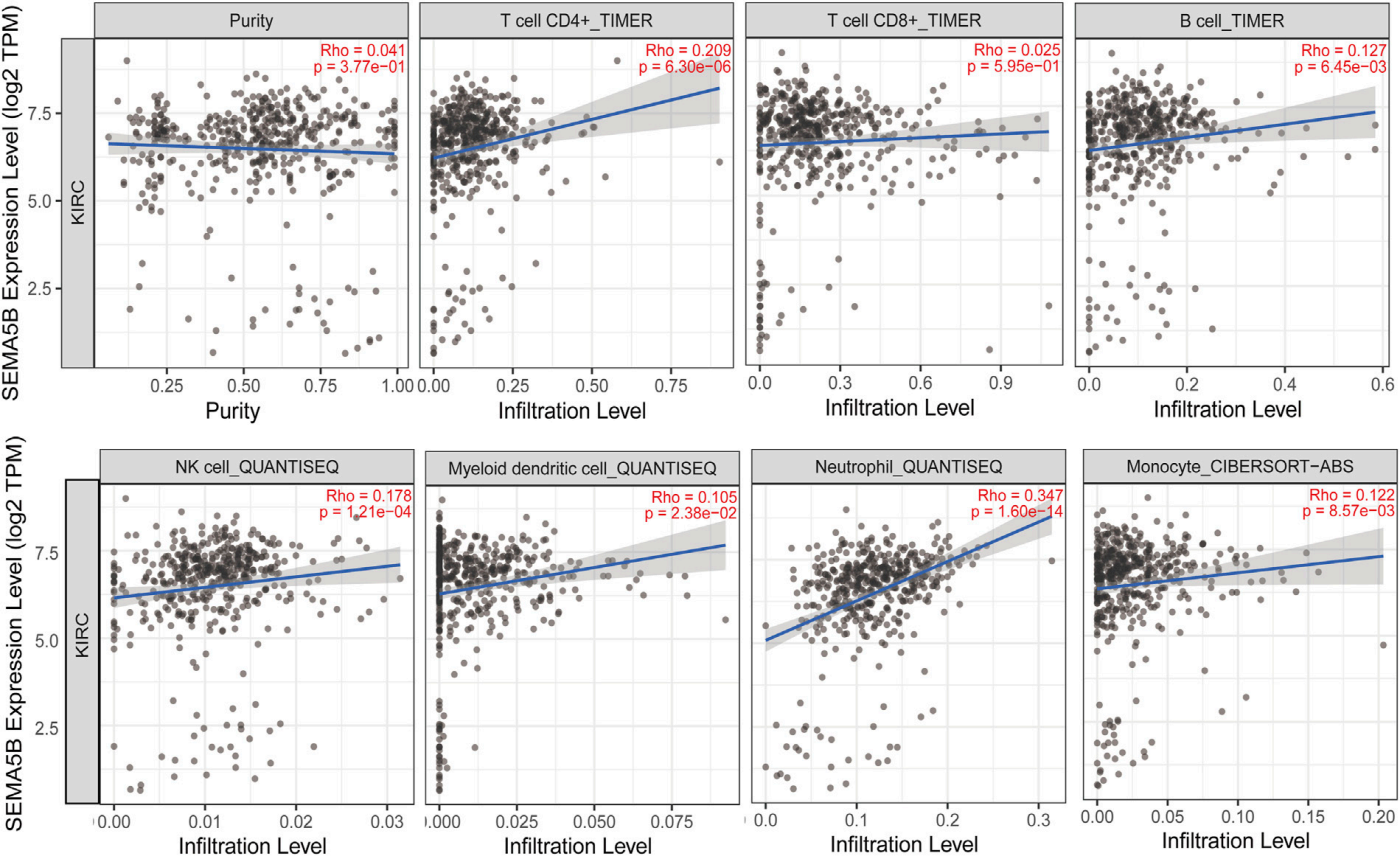
Correlation of SEMA5B expression with immune infiltration level in KIRC. The study was performed. The correlation between the SEMA5B expression and immune cells was conducted in the TIMER database and evaluated by Spearman’s correlation.

High levels of tumor-infiltrating lymphocytes (TIL) have been reported to correlate with favorable prognoses in a variety of solid organ malignancies. The relationship between tumor-infiltrating lymphocytes (TIL) and SEMA5B expression was examined. As shown in Figure 6, SEMA5B expression was significantly positive correlated with the level of CD4^+^ T cells (Rho = 0.209, *p* = 6.30e-06), B cells (Rho = 0.127, *p* = 6.45e-03), NK cells (Rho = 0.178, *p* = 1.21e-04), myeloid dendritic cells (Rho = 0.105, *p* = 2.38e-02), neutrophils (Rho = 0.347, *p* = 1.60e-14), and monocytes (Rho = 0.122, *p* = 8.57e-03). However, there was no significant correlation between the expression level of SEMA5B with tumor purity (Rho = 0.041, *p* = 3.77e-01) and the level of CD8^+^ T cells (Rho = 0.025, *p* = 5.95e-01). Together, these results demonstrate that SEMA5B could promote the recruitment of immune cells in the tumor microenvironment in KIRC.

### Immune Checkpoint Gene Analysis

**FIGURE 7:**
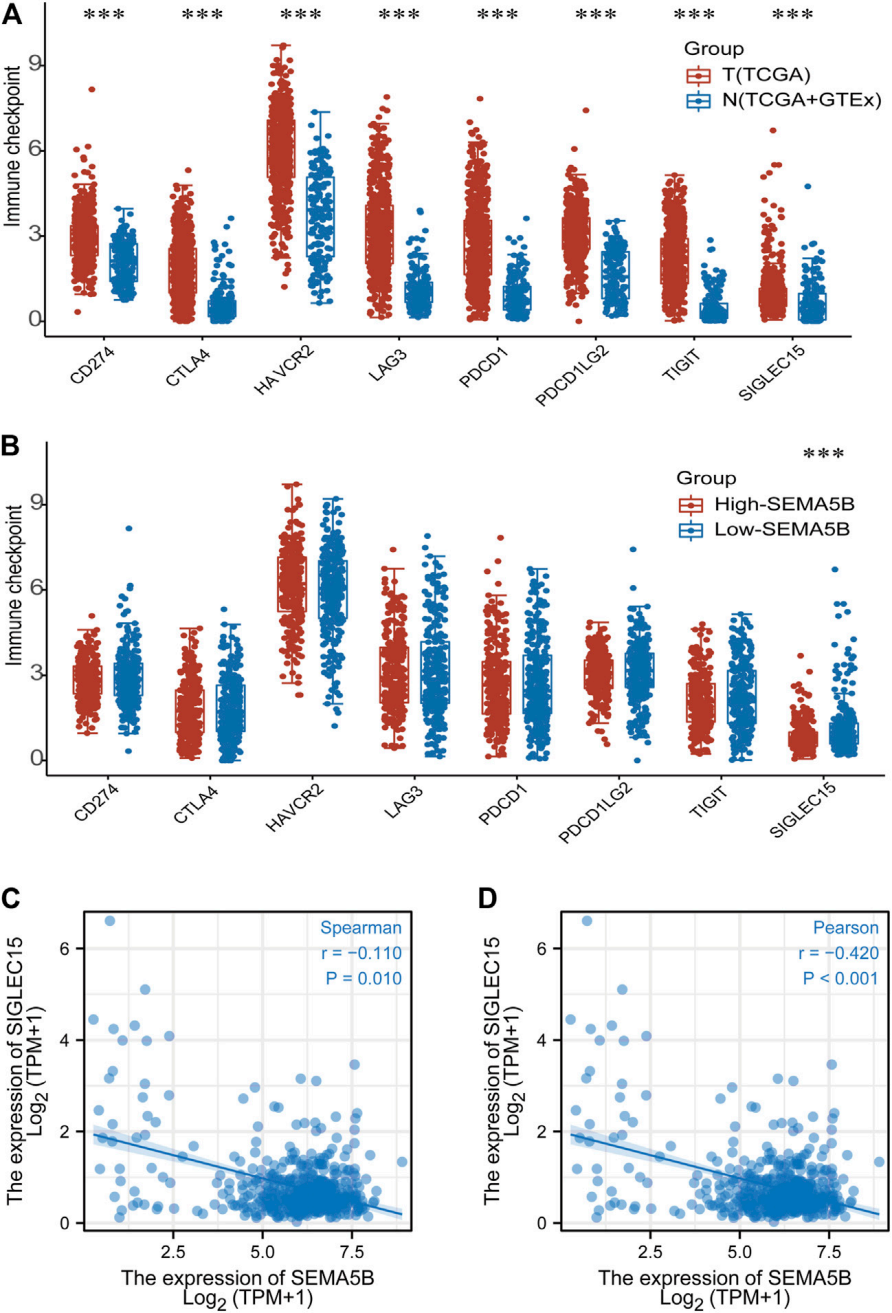
Immune checkpoint gene analysis. **(A)** The expression distribution of checkpoint genes in tumor tissues and normal tissues. **(B)** Expression of checkpoint genes in high- and low-SEMA5B expression groups. Spearman correlation **(C)** and Pearson correlation **(D)** for the relationship between the expression levels of SEMA5B and SIGLEC15. ***p < 0.001.

Different cancer types can manipulate immune checkpoint expression to evade immunosurveillance, leading to the development of immune checkpoint blockade as a successful therapeutic strategy for certain cancers. We detected the expression levels of 8 immune checkpoint-related genes (SIGLEC15, TIGIT, CD274, HAVCR2, PDCD1, CTLA4, LAG3, and PDCD1LG2). As shown in Figure7A, there were significantly higher expression of SIGLEC15, TIGIT, CD274, HAVCR2, PDCD1, CTLA4, LAG3, and PDCD1LG2 in the KIRC tissues compared to the normal tissues (all *p* < 0.05). SIGLEC15 was found to be higher in the low-SEMA5B expression group than in the high-expression group (Figure7B). Correlation analysis also exhibited a negative correlation between SEMA5B expression and that of SIGLEC15 (Figures 7C,D). These results suggested that patients with high SEMA5B expression may have better immunotherapy effects and reflect better survival.

### SEMA5B Is Correlated With MMR Gene Levels and DNA Methyltransferase Gene Expression in Humans

Previous studies have shown that DNA mismatch repair (MMR) deficiency leads to DNA replication errors (Georgakopoulos-Soares et al., 2020), higher somatic mutations, and tumorigenesis. In order to determine the potential role of SEMA5B in the prognosis of KIRC, we evaluated the association of the expression level of SEMA5B with the levels of five MMR genes. Results revealed that SEMA5B expression exhibited a positive relationship to MMR genes in KIRC (Table 4). Recently, DNA methylation has been recognized as an epigenetic modification that can affect gene expression (Tiffen et al., 2020). Also, the status change of the DNA methylation is considered to be an important factor in tumorigenesis. The correlations between SEMA5B and four DNA methyltransferases were further evaluated. Evidently, SEMA5B expression is closely related to the expression of DNMT1, DNMT2, DNMT3A, and DNMT3B in KIRC.

**TABLE 4:** Correlation analysis between SEMA5B and MMRs and DNA methyltransferase genes in TIMER and GEPIA.

Description	Gene markers	TIMER2		GEPIA	
		R	*p* value	R	*p* value
DNA MMRs	EPCAM	0.22	**1.5E-06**	0.21	**1.8e−06**
	MLH1	−0.10	**3.4E-02**	0.022	0.61
	MSH2	0.17	**3.5E-04**	0.19	**1.9e−05**
	MSH6	0.17	**1.7E-04**	0.19	**1.7e−05**
	PMS2	0.16	**4.1E-04**	0.12	**8.3e−04**
DNA methyltransferase genes	DNMT1	0.13	**3.8E-03**	0.17	**1.1e−05**
	DNMT2	0.009	0.846	0.19	**1.5e−05**
	DNMT3A	0.20	**1.3E-05**	0.22	**2.3e−07**
	DNMT3B	0.12	**8.4E-03**	0.16	**3.1e−05**

Note: Data in bold indicates *p* < 0.05.

### SEMA5B DNA Methylation Analysis

**FIGURE 8:**
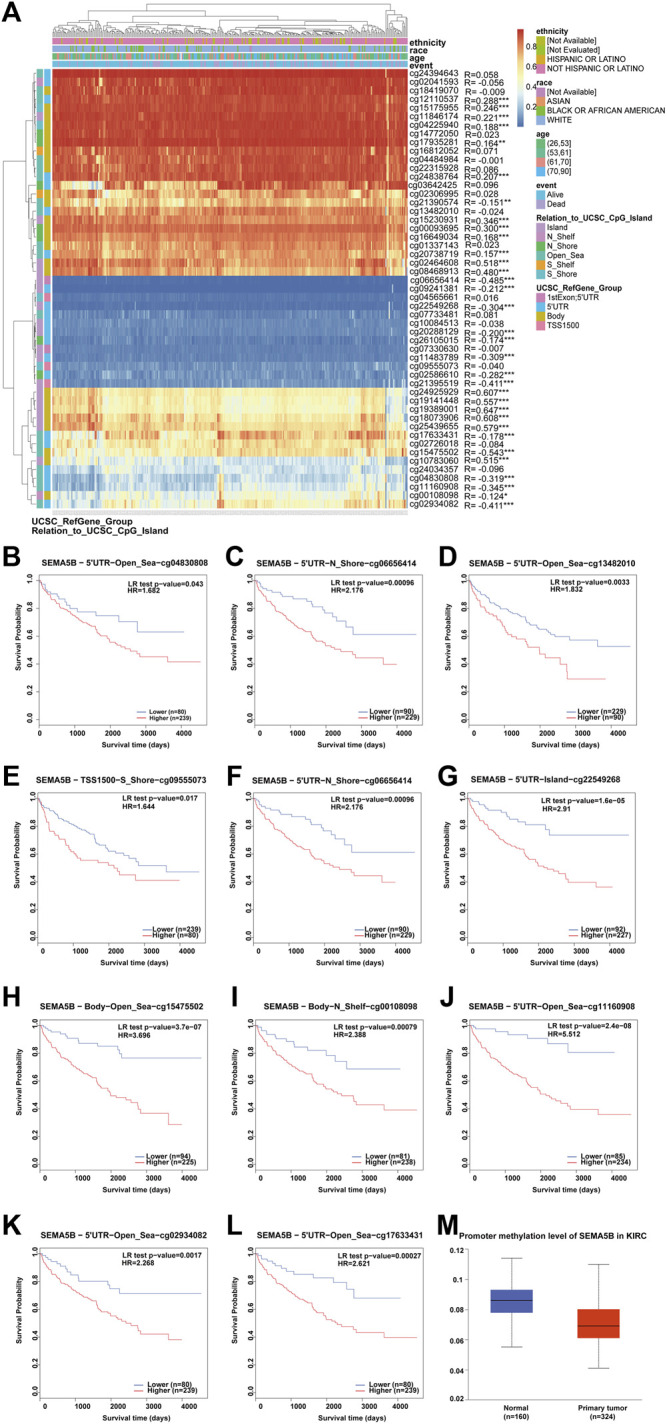
Analysis of SEMA5B methylation in KIRC. **(A)** Heat map of the methylation level of the SEMA5B gene. The correlations between SEMA5B methylation and its expression were analyzed. *p* < 0.05 was considered statistically significant. *p < 0.05, **p < 0.01, ***p < 0.001. **(B–J)** Survival analysis based on methylation at multiple sites. **(M)** The promoter methylation level of SEMA5B in KIRC.

To further address whether SEMA5B expression might be influenced by DNA methylation states in KIRC, we utilized MethSurv tool to visualize the correlation between gene expression and methylation sites. We identified 33 significantly differentially methylated CpG sites. Notably, methylation in these sites correlated inversely with gene expression: cg06656414, cg21390574, cg09241381, cg22549268, cg20288129, cg26105015, cg11483789, cg02586610, cg21395519, cg17633431, cg15475502, cg04830808, cg11160908, cg00108098, and cg02934082 (Figure 8A). Survival analysis revealed that hypermethylation at cg04830808, cg06656414, cg13482010, cg09555073, cg21390574, cg22549268, cg15475502, cg00108098, cg11160908, cg02934082, and cg17633431 in the SEMA5B promoter was correlated with a poor prognosis (Figures 8B–L). In addition, as shown in Figure 8M, methylation levels in the SEMA5B-promoter region were significantly higher in normal subjects than in KIRC patients. These results were consistent with our previous survival analysis (Figures 3J, K), suggesting that the SEMA5B gene methylation in KIRC correlates with the progression of this type of cancer.

### Expression of m6A RNA Methylation Regulators in KIRC

**FIGURE 9:**
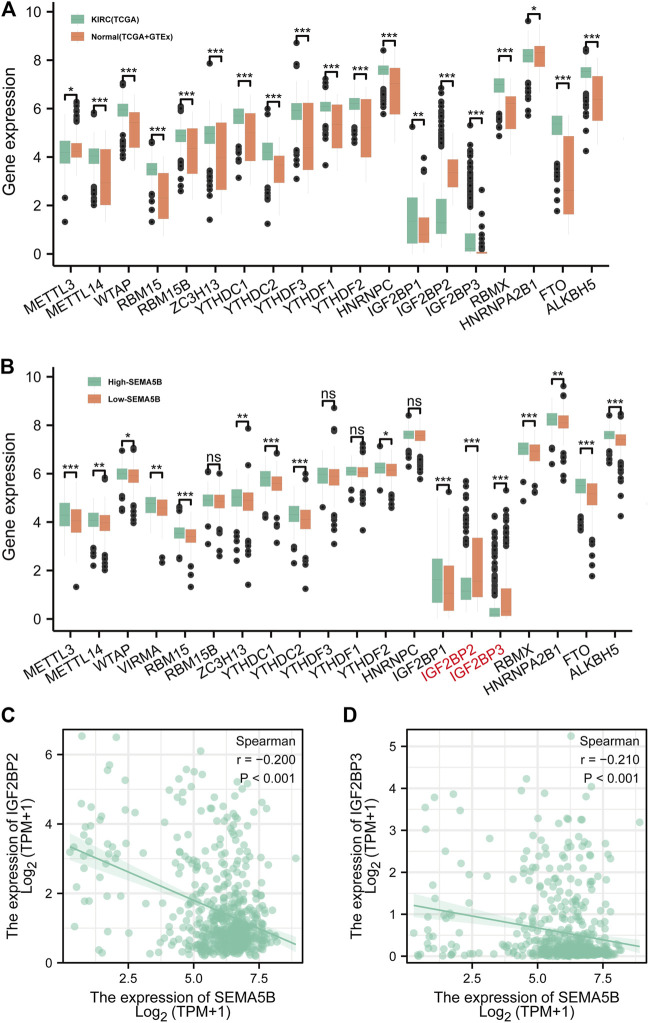
The correlation between the SEMA5B expression and m6A regulators in KIRC. **(A)** m6A regulators expression between KIRC tissues and normal tissues according to the TCGA and GTEx. **(B)** m6A regulators expression between high- and low-SEMA5B expression groups of KIRC. Spearman correlation for the relationship between the expression levels of SEMA5B and IGF2BP2 **(C)**, and IGF2BP3 **(D)**. ns, *p* ≥ 0.05; *, *p* < 0.05; **, *p* < 0.01; ***, *p* < 0.001.

Numerous recent studies have shown that m6A modification plays a key role in various types of carcinogenesis (Panneerdoss et al., 2018; Zheng et al., 2021). In current study, the following 19 common m6A regulators were studied: METTL3, METTL14, WTAP, RBM15, RBM15B, ZC3H13, YTHDC1, YTHDC2, YTHDF3, YTHDF1, YTHDF2, HNRNPC, IGF2BP1, IGF2BP2, IGF2BP3, RBMX, HNRNPA2B1, FTO, and ALKBH5. Based on the TCGA data cohort and the normal tissue of the GTEx dataset, we found that all the 19 m6A regulators were differentially expressed between KIRC tissues and normal tissues, including three downregulated genes (METTL3, IGF2BP2, and HNRNPA2B1) and 16 upregulated genes (METTL14, WTAP, RBM15, RBM15B, ZC3H13, YTHDC1, YTHDC2, YTHDF3, YTHDF1, YTHDF2, HNRNPC, IGF2BP1, IGF2BP3, RBMX, FTO, and ALKBH5) (Figure 9A). In order to further study the expression correlation between SEMA5B and m6A regulators, we conducted a comparative analysis on the expression of m6A regulators with high and low SEMA5B expression of KIRC. Results showed that IGF2BP2, IGF2BP3 expression were significantly decreased in high-SEMA5B group than the low-SEMA5B expression group (Figures 9B–D). These results suggest that m6A regulators may involve in the expression of SEMA5B in KIRC.

### GSEA Analysis of SEMA5B Expression

To find enriched pathways related to SEMA5B and to identify potential mechanism of SEMA5B’s biological role in KIRC, we then performed the GSEA analysis (S1). Results of GSEA showed SYSTEMIC_LUPUS_ERYTHEMATOSUS and NABA_ECM_REGULATORS were prominently enriched in the low-SEMA5B expression phenotype.

## Discussion

Although early study has reported SEMA5B upregulation in clear cell renal carcinoma (ccRCC) and suggested the role of SEMA5B in tumor growth (Kundu et al., 2020), there is a lack of comprehensive studies on the clinical role of SEMA5B. Therefore, we used bioinformatics analysis of multiple online databases to investigate and obtain more comprehensive insights into the possible roles of SEMA5B in KIRC.

In this study, we observed prominent upregulated mRNA and protein expression of SEMA5B in KIRC and clear cell renal carcinoma cell lines. Decreased SEMA5B expression was correlated with older age, male, higher T stage and N stage, advanced histological grade and pathological stage, and shorter OS and DSS. We also revealed and validated a marked diagnostic capability of SEMA5B for KIRC. The co-expression analysis further indicated that SEMA5B and its co-expressed genes may act as good prognosis markers for KIRC. Furthermore, SEMA5B and its co-expressed genes were specifically enriched in small molecule catabolic process, transport of small molecules, positive regulation of inflammatory response, and diseases of signal transduction by growth factor receptors and second messengers. They may also involve in crosslinking in the extracellular matrix (ECM). Finally, we found that SEMA5B expression was correlated with immune checkpoint, DNA repair and methylation, immune cell infiltration, and various m6A RNA methylation regulators. Thus, our study elucidates the clinical value of SEMA5B in KIRC, complements the results of previous studies (Hirota et al., 2006; Kundu et al., 2020), and provides a different perspective on how SEMA5B affects cancer progression and metastasis as well as provides insights for improving cancer immunotherapy in the future.

Although the effects of SEMA5B were reported more than 30 years ago, the links between SEMA5B and human cancer has remained elusive. In this study, we conducted a comprehensive differential expression analysis of SEMA5B in normal and tumor from multiomics data integration and analysis in specific cancer. Results show that the mRNA expression level of SEMA5B is upregulated in a variety of tumors. Second, we found and confirmed that SEMA5B exhibited a significantly higher mRNA and protein expression profiles in KIRC tumor tissues and cell lines through multiple databases. SEMA5B was reported to upregulate in KIRC and effective downregulation of its expression levels in RCC cells significantly attenuated RCC cell viability (Hirota et al., 2006). Upregulated SEMA5B also significantly promoted proliferation in HK2 cells (Kundu et al., 2020). In summary, it confirms that SEMA5B expression was significantly higher in tumors rather than normal tissues in KIRC. A high expression level of KIRC may involve in the development of KIRC. A recent study has shown that the upregulated SEMA5B was closely related to the prognosis of gastric adenocarcinoma (Cao et al., 2021). In our study, we also found that the expression of SEMA5B was significantly correlated with the OS and DSS of KIRC. Previous research suggested that the upregulated SEMA5B may be a candidate marker for the diagnosis of KIRC (Hirota et al., 2006). In our study, based on the mRNA expression level in TCGA, the ROC curve for SEMA5B discrimination of KIRC diagnosis had an AUC of 0.928. Moreover, we demonstrated that SEMA5B showed strong diagnostic ability at different stages of tumor diagnosis. In addition, we validated our results on the inference of four different datasets in GEO, and found consistent and significant diagnostic efficiency. All of these strongly suggest that SEMA5B is a convincing biomarker for KIRC diagnosis.

Genes which are co-expressed have a higher probability of having related functions than those which are not co-expressed (Farahbod and Pavlidis, 2020). We conducted the co-expression analysis and explored the potential functions and mechanisms involving SEMA5B in KIRC. Our findings demonstrate that SEMA5B was highly positively correlated with the top six co-expressed genes (PDIA5, ACAD11, SLC25A34, EEFSEC, IFT122, and SLC23A3), and five of these genes served as a protective factor for the favorable survival of KIRC. However, some of these genes have been reported to act as poor prognosis in malignancy (Kho et al., 2021; Zhang et al., 2021). To further explore the potential oncogenic mechanism of SEMA5B action, we conducted the GO and KEGG analyses of the co-expressed genes. The enrichment analysis indicated that SEMA5B may be involved in several KIRC-related pathways, including nuclear receptors meta-pathway, NABA ECM AFFILIATED, and constitutive androstane receptor pathway. We further investigated the function of SEMA5B and the probable mechanism underlying the effects of SEMA5B on the progression and metastasis KIRC based on GSEA. As there is little literature on SEMA5B, with the performance of GSEA, we only found that _SYSTEMIC_LUPUS_ERYTHEMATOSUS and NABA_ECM_REGULATORS were significantly enriched in the SEMA5B low-expression phenotype.

Notably, SEMA5B may be involved in small molecule catabolic process, transport of small molecules, positive regulation of inflammatory response, and diseases of signal transduction by growth factor receptors and second messengers. It is well accepted that adhesion to ECM is a crucial step in cancer progression; the ECM components such as collagen, fibroblasts, and their associated signaling molecules contribute to tumor cell proliferation, migration, and invasion in various cancers (Brassart-Pasco et al., 2020; Moreira et al., 2020). In brief, those results exhibited that SEMA5B may play a crucial role in KIRC through crosslinking in the extracellular matrix (ECM).

In many cancer types, an immune infiltrate within the tumor is typically associated with a better prognosis and with response to immunotherapy (Robertson et al., 2017). In the present study, the abundance of infiltration by multiple immune cells (such as, CD4^+^ T cells, B cells, NK cells, Myeloid dendritic cells, neutrophils, and monocytes) was found to significantly positively correlate with the expression of SEMA5B. The role of CD4^+^ T cells is increasingly being studied that stromal infiltration of CD4^+^ T cells in cancers is associated with better OS and disease-specific survival (Soo et al., 2018; Niemeijer et al., 2020). NK cells have powerful antitumor effects. Similarly, NK cell infiltrates in primary colorectal, gastric, and lung cancer proved to be correlated with better patient survival outcomes (Malladi et al., 2016). Tumor-infiltrating dendritic cells are potent antigen-presenting cells (Mathios et al., 2016). Neutrophils are proven to be associated with better prognosis in different cancer types (Qu et al., 2018). Moreover, upon infiltration, monocytes differentiate and promote tumor cell death through an as-yet unknown mechanism (Maximov et al., 2019). Therefore, overexpressed SEMA5B appeared to enhance tumor immunity, synergistically enhance immune cell infiltration of tumor, and further boost the antitumor immune response, and finally inhibit tumor progression.

The importance of immune‐checkpoints has been increasingly recognized in tumor immunology. During tumorigenesis, various immune checkpoints are induced to create an immunosuppressive TME for escaping immune surveillance (Quail and Joyce, 2013). We found immune checkpoints genes including SIGLEC15, TIGIT, CD274, HAVCR2, PDCD1, CTLA4, LAG3, and PDCD1LG2 were highly expressed in KIRC. Previous studies have reported that SIGLEC15 as an immune suppressor inhibits T cell proliferation and activation *in vitro* and *in vitro* (Wang et al., 2019). In addition, they found SIGLEC15 deficiency promoted T cell responses, better tumor control, and overall survival in a mouse melanoma model. In this study, we found that SEMA5B levels showed a negative correlation with SIGLEC15 in KIRC, suggesting that SEMA5B may effect of SIGLEC15 on tumor immunity. Combined with the previous findings, we speculated that the elevated SEMA5B may interfere with SIGLEC15 expression through some unknown mechanism, thus affecting the immune effect of cytotoxic T cells and ultimately prolonging the survival of tumor patients. It suggests that SIGLEC15 is highly likely to be a potential therapeutic target of KIRC. However, the specific mechanism still needs further in-depth study.

Previous literature indicates that mutations or defects in the MMR genes (MLH1, MSH2, MSH6, PML2, and EPCAM) can lead to the accumulation of genetic errors, resulting in genomic or microsatellite instability, which contributes to the development of tumors (Armaghany et al., 2012). In addition, the function loss of those genes brings about a mutator phenotype which causes an increasing tumor risk (Jiricny, 2006). Our results show that SEMA5B expression was positively related to the expression of MMRs in KIRC. The relationship between DNA methyltransferases (DNMTs) and tumorigenesis has been widely discussed. Previous studies have shown that DNA methylation is a common epigenetic feature of cancer. (Manoochehri et al., 2020; Yin et al., 2020). In this study, we found a significantly positive correlation between the expression of SEMA5B and DNMTs levels in KIRC. Moreover, we found a correlation between hypermethylation at multiple sites of SEMA5B and a poor prognosis in KIRC. In general, high levels of promoter methylation tend to reduce gene expression or silence the gene (Jones, 2012). Our findings show that the degree of methylation in the SEMA5B promoter region was significantly lower in KIRC compared to normal people. Thus, our findings indicate that gene-related modifications may have little influence on the gene expression of SEMA5B, while factors that reduce the expression of SEMA5B will more or less affect the survival of patients, which further demonstrates that SEMA5B is indeed a protective factor of KIRC.

Although our current study has conducted in-depth exploration of the role of SEMA5B in KIRC, there are still some limitations. First, despite using multiple database data, however, it was not validated in our own clinical samples. Thus, further experimental verifications are necessary. Second, the prognostic value of SEMA5B in KIRC also needs further verification. Third, this study only focuses on the expression, diagnosis, and prognosis of SEMA5B, the mechanisms by which SEMA5B promotes tumor progression and metastases in KIRC need further elucidation.

## Conclusion

In conclusion, our study indicated that SEMA5B is significantly upregulated and is associated with immune infiltration in KIRC. SEMA5B can serve as a favorable prognostic factor and a novel diagnostic biomarker for KIRC. In addition, SEMA5B may involve in crosslinking in the extracellular matrix (ECM) and correlates with the abundance MMR genes, DNMTs, and m6A regulators in KIRC. Therefore, we hypothesized that SEMA5B may be promising molecular targets for the early diagnosis, a potential prognostic biomarker and targeted therapy of KIRC.

## Data Availability

The original contributions presented in this study are included in the article/Supplementary Material; further inquiries can be directed to the corresponding author.
