# Supplementary figures and images for: Prognostic and Diagnostic Values of Semaphorin 5B and Its Correlation With Tumor-Infiltrating Immune Cells in Kidney Renal Clear-Cell Carcinoma
Source: Front Genet. 2022 Apr 11;13:835355. doi: 10.3389/fgene.2022.835355 (PMC9035641; doi:10.3389/fgene.2022.835355)

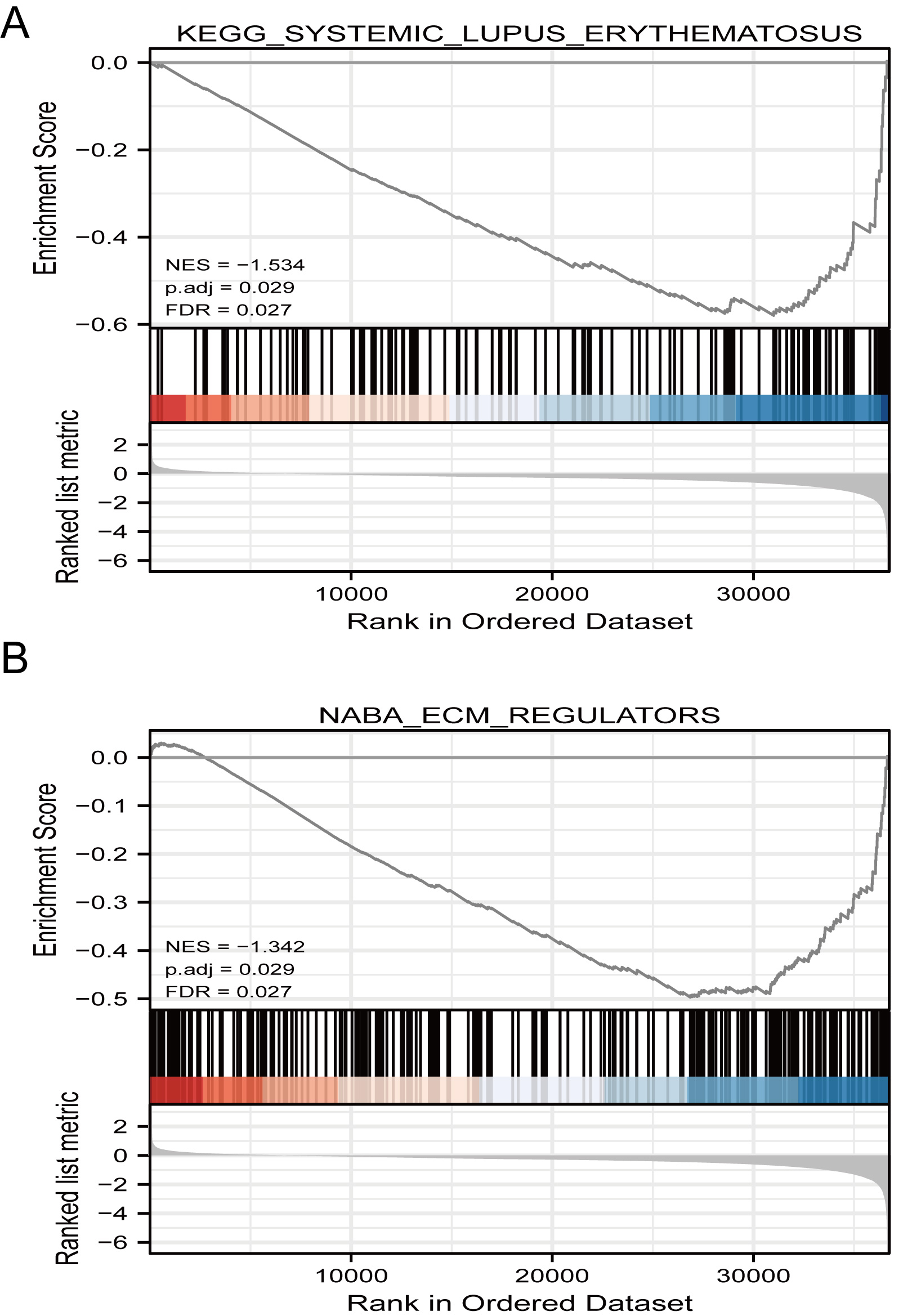

Supplement: Supplementary file 1 [file Image1.JPEG]
